# Auditory Development of Young Children with Profound Hearing Loss, Cochlear Implants, and Congenital CMV Infection

**DOI:** 10.3390/jcm13226734

**Published:** 2024-11-08

**Authors:** Piotr H. Skarzynski, Anita Obrycka, Aleksandra Kolodziejak, Artur Lorens, Elzbieta Gos, Rita Zdanowicz, Henryk Skarzynski

**Affiliations:** 1 Teleaudiology and Screening Department, World Hearing Center, Institute of Physiology and Pathology of Hearing, 02-042 Warsaw, Poland; e.gos@ifps.org.pl (E.G.); r.zdanowicz@ifps.org.pl (R.Z.); 2 Institute of Sensory Organs, 05-830 Kajetany, Poland; 3 Implant and Auditory Perception Department, World Hearing Center, Institute of Physiology and Pathology of Hearing, 02-042 Warsaw, Poland; a.obrycka@ifps.org.pl (A.O.); a.lorens@ifps.org.pl (A.L.); 4 Otorhinolaryngosurgery Clinic, World Hearing Center, Institute of Physiology and Pathology of Hearing, 02-042 Warsaw, Poland; h.skarzynski@ifps.org.pl

**Keywords:** CMV infection, cochlear implants, auditory development

## Abstract

**Background/Objectives:** The aim of this study was to assess auditory development in young children with profound hearing loss, cochlear implants (CIs), and congenital cytomegalovirus (cCMV) infection and to determine the effect of comorbidities on their development. **Methods:** The study group (cCMV group) consisted of 47 CI children—18 girls and 29 boys—who had been diagnosed as having prelingual hearing loss due to cCMV infection (with or without comorbidities); the mean age at CI activation was 15.2 months (range: 9.7–23.8; SD = 3.5). The reference group (no cCMV) consisted of 117 similar children (57 girls and 60 boys) who had profound sensorineural hearing loss not caused by cCMV infection; they had no comorbidities. The mean age at CI activation in the second group was 14.3 months (range: 7.9–23.5; SD = 4.0). Auditory development in all children was assessed with the LittlEARS Auditory Questionnaire (LEAQ) at CI activation and at about 1, 5, 9, 14, and 24 months of CI use. **Results:** The mean LEAQ total score increased over a similar time frame from 9.8 pts to 28.9 pts in the cCMV group without comorbidities, from 4.5 pts to 18.5 pts in the cCMV group with comorbidities, and from 9.2 to 31.6 pts in the reference group with no cCMV infection. **Conclusions:** Early cochlear implantation in children with sensorineural hearing loss due to congenital CMV infection and no comorbidities promotes their early auditory development in a similar way to children without cCMV infection.

## 1. Introduction

Cytomegalovirus (CMV) is a virus belonging to the *herpes* family, which also includes the Varicella virus, the Epstein–Barr virus, and Herpes Simplex. The virus is very common, although many people are unaware of their infection and pass the disease on asymptomatically [1,2,3]. The disease is diagnosed by PCR tests of the patient’s urine or saliva or serological testing measuring IgG and IgM antibodies in venous blood [4,5,6,7]. Treatment includes a diet of easily digestible food and pharmacotherapy, such as antipyretics or, in immunocompromised individuals, ganciclovir, which inhibits virus replication. A complete cure of CMV is not possible, as the virus becomes dormant after the acute phase and has the ability to reactivate [8,9].

Congenital CMV infection is one of the most common intrauterine infections. Approximately 5–7 of 1000 newborns are infected with CMV, which they catch from their mother during pregnancy [10]. Hormonal changes and compromised immunity increase a pregnant woman’s risk of infection [11,12]. Pregnant women with primary CMV infection have about a 40% risk of transmitting the infection to their fetus; if the infection occurs in the third trimester, the risk of infecting the baby rises to almost 70% [5,11,12,13]. Although infection with the virus is widespread, routine screening is not carried out and consequently many children are living with the long-term consequences of infection [14]. Complications of cCMV can include intellectual disability, epilepsy, hearing loss, cerebral palsy, visual impairment, gastrointestinal ulcers, and microcephaly. Symptoms appear in 10–15% of infected children, but up to 90% of them will develop long-lasting sequelae. However, complications can manifest later, even at school age. It is therefore very important to monitor and control the health of children born with CMV infection [15,16].

Hearing loss caused by congenital CMV infection is sensorineural, can be unilateral or bilateral, and the degree of hearing loss can range from mild to complete deafness [17,18]. Diagnosis of hearing loss is complicated by the fact that only half of CMV-related hearing loss occurs immediately after birth; the other half may occur up to the age of 6 years. Over time, untreated hearing loss can affect a child’s ability to develop language, social skills, and communication. It is therefore advisable to continue monitoring the hearing of children with congenital CMV infection, even if the screening result after birth is normal. Hearing screening every 6 months is recommended for this group of patients [19,20]. For children who are diagnosed with hearing loss, two hearing solutions are possible. In mild to severe cases, hearing aids can be effective. However, in cases of severe to profound hearing loss, if children do not benefit from a trial with hearing aids, a cochlear implant (CI) should be considered [21,22,23,24].

Some research has already looked at the auditory development of children with cCMV infection who have received a CI and compared their hearing ability to control groups. Yamakazi and colleagues [25] found poorer social and language development and weaker word discrimination skills in their CMV children, who, as it happened, also had a high incidence of comorbidities. Another study by Ciorba and colleagues [26] found slower progress in speech perception and production in a cCMV group compared to a control group. Such deficits have been attributed to cognitive impairments associated with cCMV. On the other hand, studies by Iwasaki and coworkers [27] and Matsui and collaborators [28] found no statistical differences between the auditory development of children with and without cCMV infection.

Given the disparity, the aim of this study was to make an assessment of early-implanted children and their early auditory development in a group of CI children who had profound hearing loss, CIs, and congenital CMV. Additionally, we investigated the effect of comorbidities on early auditory development in these children.

## 2. Materials and Methods

### 2.1. Study Design

This retrospective case review study was conducted at the Institute of Physiology and Pathology of Hearing, a tertiary ENT referral center in Poland. The study was performed in accordance with the ethical standards of the Institute and the 1964 Declaration of Helsinki. The study protocol was reviewed and approved by the Institutional Review Board (IFPS:KB/Statement 2/2023). Formal consent is not required for this type of retrospective study.

### 2.2. Patients

The children recruited for the study were implanted based on a standard widely accepted clinical protocol [29]. The additional eligibility criteria for the study were as follows: confirmation of congenital CMV infection: 29 children underwent a PCR urine or saliva test while in the neonatal unit and 18 children underwent serological testing (at the pediatric center) to determine their levels of IgM and IgG antibodies (before the age of 6 months) (cCMV-related diagnostic information was collected through a medical interview before CI); air conduction thresholds of 90 dB or more for 500 Hz tone burst- and click-evoked ABR; and age at cochlear implantation less than 2 years.

The study group (cCMV group) consisted of 47 unilaterally implanted children—18 girls and 29 boys. The mean age at CI activation was 15.2 months (min = 9.7; max = 23.8; SD = 3.5). The reference group (no cCMV group) was selected from a cohort of 122 children without comorbidities tested for a LittlEARS validation study [30]; from that group, 5 children who had cCMV and hearing loss were excluded, leaving 117 children (57 girls and 60 boys). All 117 children had no CMV-related basis for their profound sensorineural hearing loss. They were all implanted unilaterally before the age of 2 years; the mean age at CI activation was 14.3 months (min = 7.9; max = 23.5; SD = 4.0). Details of both groups are given in Table 1.

### 2.3. Variables

Auditory development in both groups was assessed with the LittlEARS Auditory Questionnaire (LEAQ) [31,32]. It was designed and validated to assess early auditory development in children with normal hearing or in children with hearing loss [30,32,33]. The tool is completed by parents, who answer “yes” or “no” to a set of questions which reflect their child’s auditory behavior in everyday situations. The LEAQ total score can range from 0 to 35 points, where the age-dependent norm of 33 points reflects the auditory development of a 2-year-old child with normal hearing [32,33].

We chose this questionnaire because it allows for a comprehensive assessment of a child’s hearing abilities as they advance with age. Other methods allow only for behavioral assessment of sound detection (e.g., Visual Reinforcement Audiometry) or can only be used with children older than 3 years (e.g., speech tests). Moreover, the results of electrophysiological assessments are difficult to directly translate into stages of auditory development.

Considering the above-mentioned challenges in assessment of early auditory development, given the excellent psychometric properties of the LEAQ [30,32,33] and the confirmation of its validity through electrophysiological measures [34], the choice of this tool seems highly justified.

The LEAQ was completed at CI activation and at around 1, 5, 9, 14, and 24 months of CI use, although each test point varied across subjects (except for CI activation). Moreover, there were some missing data points—not all patients completed an evaluation at all time-points. However, to compare auditory development between groups, one needs to have a complete set of LEAQ results at each time point. So, to deal with these complications, we calculated a quadratic function for each child using the total score as the dependent variable and the duration of CI use as the independent variable. In this way, we could estimate the LEAQ results for each child at exactly 1, 3, 6, 9, and 12 months after CI activation. The procedure is described in Obrycka et al. [30].

### 2.4. Statistical Methods

Data were analyzed using Statistica version 12. Two-way repeated-measures ANOVA was used to determine (1) the effect of time on auditory development and (2) whether development differed between the study group and the reference group. The hypothesis of a normal data distribution was tested using a Shapiro–Wilk test, variance homogeneity was assessed with Levene’s test, and sphericity for repeated measures was assessed with Mauchly’s test. Due to violation of the sphericity assumption, the multivariate test method Wilk’s Lambda was used with modifications to the degrees of freedom to obtain a valid F-ratio. The level of significance was set at *α* = 0.05.

## 3. Results

**Figure 1:**
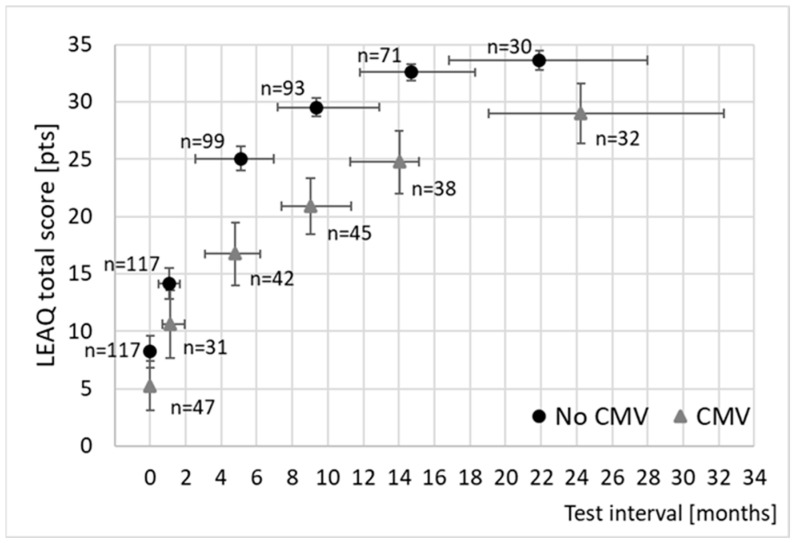
Mean LEAQ total scores for the study group (triangles) and the reference group (circles). The vertical whiskers are 95% confidence intervals; horizontal whiskers correspond to the time range for each test.

Figure 1 shows the LEAQ total scores for both the study group (cCMV) and the reference group (no cCMV) as a function of time. The mean LEAQ total score for the CMV group increased from 5.2 points at CI activation to 24.7 pts at around 14 months of CI use, while in the no-CMV group it increased from 8.2 to 32.6 points over the same time. Statistically, however, matters were complicated because the test intervals varied not only within each group but also between them (see the whiskers in Figure 1); the number of children also varied across intervals (see data labels in Figure 1). To formally analyze the differences between groups and to provide a complete data set for a repeated-measures design, the estimation described in the preceding section was used. Initially, the difference between the cCMV and no-cCMV groups was investigated. Further data analysis was conducted to determine if the presence of comorbidities was significant.

**Figure 2:**
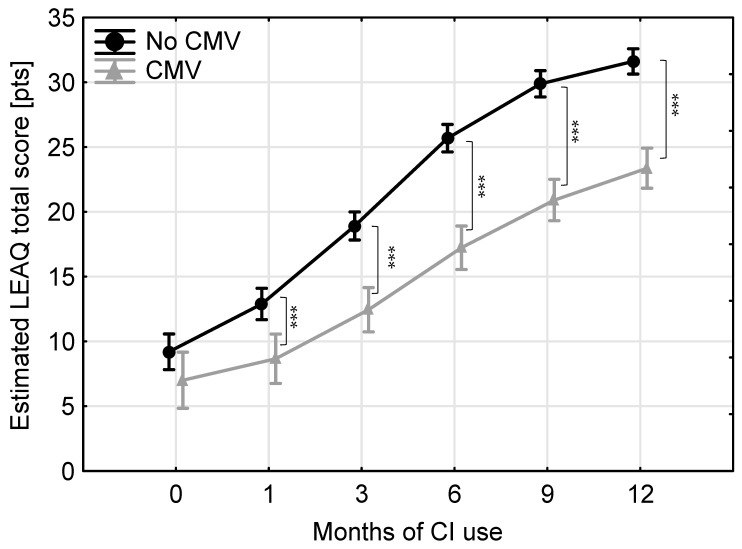
Average estimated LEAQ total scores at identical points in time for all children. Triangles—study group; circles—reference group; whiskers—95% confidence intervals, asterisks—significant differences: *** *p* < 0.001.

The mean estimated LEAQ total scores for identical points in time for each child are shown in Figure 2. Repeated-measures ANOVA showed that both groups improved significantly with duration of CI use (*F*(5,158) = 157.88, *p* < 0.001, Wilk’s Lambda = 0.17). Repeated-measures ANOVA also revealed an interaction between duration of CI use and test group (*F*(5,158) = 5.97, *p* < 0.001, Wilk’s Lambda = 0.84). Univariate analysis was used to make post hoc comparisons between the groups and showed that no-CMV children performed significantly better than children from the CMV group at all test intervals (except for at CI activation). The differences between the CMV group and the no-CMV group were as follows: 2.2 pts at CI activation (*t*(162) = 1.69, *p* = 0.09); 4.3 pts at 1 month of CI use (*t*(162) = 3.70, *p* = 0.0003); 6.5 pts at 3 months (*t*(162) = 6.31, *p* < 0.0001); 8.5 pts at 6 months (*t*(162) = 8.40, *p* < 0.0001); 9.0 pts at 9 months (*t*(162) = 9.37, *p* < 0.0001); and 8.2 pts at 12 months of CI use (*t*(162) = 8.90, *p* < 0.0001).

**Figure 3:**
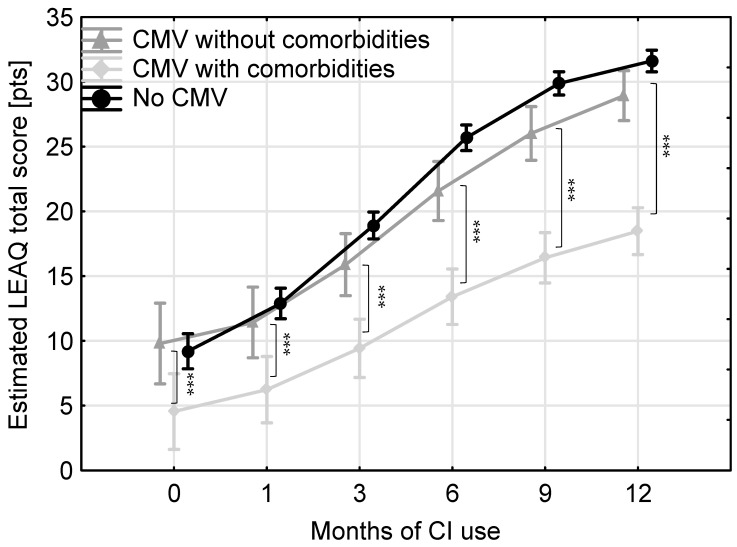
The average estimated LEAQ total scores for the no-CMV group (circles), the CMV group without comorbidities (triangles), and the CMV group with comorbidities (diamonds). Whiskers indicate 95% confidence intervals; asterisks indicate significant differences: *** *p* < 0.001.

In the cCMV group, 22 of the 47 children were identified as having no comorbidities (Table 1). To investigate the effect of comorbidities, we compared auditory development in those with and without comorbidities. When the presence of comorbidities was taken as a between-subjects factor, it revealed a significant effect of CI use (*F*(4,41) = 39.48, *p* < 0.001, Wilk’s Lambda = 0.17), and children with comorbidities showed significantly lower levels of auditory development at all intervals (Figure 3). The differences were 5.25, 5.19, 6.46, 8.16, 9.60, and 10.34 pts at 0, 1, 3, 6, 9, and 12 months of CI use, respectively, and were significant at *p* < 0.001.

Figure 3 compares the results of the CMV subgroup with comorbidities, the CMV group without comorbidities, and the no-CMV group. There was a significant effect of CI use (*F*(5,157) = 107.76, *p* < 0.001, Wilk’s Lambda = 0.23) and a significant interaction effect between time of CI use and test group (*F*(10,314) = 3.73, *p* < 0.001, Wilk’s Lambda = 0.80). Post hoc comparisons using the Tukey HSD test showed that the level of auditory development was comparable across all intervals for the no-CMV group and the CMV subgroup without comorbidities (*p* = 0.22), whereas scores for the CMV subgroup with comorbidities were significantly lower than the other two (*p* = 0.00002).

## 4. Discussion

On a global scale, cochlear implantation (CI) stands as a highly effective therapeutic option for children with severe-to-profound sensorineural hearing loss. Nonetheless, the results of CI can vary significantly from one child to another. Many factors have been identified as affecting CI outcomes, including etiology, the age at implantation, unilateral or bilateral implantation, the chosen mode of communication, the effectiveness of parent–child interaction, socioeconomic factors, the presence of associated symptoms, as well as inner ear anomalies or deficiencies in the cochlear nerve. For these reasons, isolating the causal factors associated with congenital cytomegalovirus (cCMV) infection is difficult. One systematic review pointed to seven studies that showed worse performance after cochlear implantation in cCMV children compared to no-cCMV children, although there were also five studies where the differences were non-significant [35]. The review found large variability in the factors affecting outcomes, the main ones being age at implantation and the presence of associated symptoms considered as comorbidities.

The present work investigated auditory development in a more homogeneous group of early-implanted CI children with cCMV infection. The children were implanted around 14 months of age. As far as we know, there is no other study on a group of early-implanted children with CMV. Our initial results have shown that children without cCMV performed significantly better after implantation than children with cCMV. However, when the cCMV children were divided into “comorbidity” and “no-comorbidity” groups, the results indicated that the poorer performance of the cCMV group compared to the no-cCMV group was due to the significantly lower performance of the comorbidity group rather than the no-comorbidity group.

In our cCMV group, there were 22 children who had no comorbidities. We were therefore able to compare the results of the cCMV subgroup with comorbidities, the cCMV group without comorbidities, and the no-cCMV group (Figure 3). Our results show that, across all test intervals, the level of auditory development was comparable for the no-cCMV group and the cCMV subgroup without comorbidities, but the scores of those in the cCMV subgroup who had comorbidities were significantly lower. This finding is in line with the conclusion of a systematic review [35] that poorer performance in cCMV children can be attributed to cCMV-related comorbidities.

Our results are also in line with those reported by Iwasaki et al. [27], who analyzed early auditory development in more than 1000 children with a CI, 18 of whom had congenital CMV infection. In this case, there were no comorbidities in either the cCMV group or the group without cCMV. Their study showed no differences in the auditory development of the cCMV and no-CMV children. Matsui et al. [28] studied five children with congenital CMV and seven children with GJB2 mutation who all underwent cochlear implant surgery. In their study, the mean age at cochlear implantation was 39.2 months for the CMV group, which was older than in our study. Using IT-MAIS and MUSS, they demonstrated a steady improvement in patient scores; however, the scores of patients who had intellectual disability were significantly lower. In a study by Yoshida and colleagues, 4 children with congenital CMV and 17 no-CMV children were tested [36]. In this case, the mean age at implantation was 2.6 yrs, meaning that these children were much older than in our work. Yoshida and colleagues found that from implantation to 12 months later, three of the four children with cCMV had IT-MAIS scores close to the maximum, comparable to the results of the no-cCMV children. Again, this result is similar to our findings.

All three studies cited above used relatively small numbers of cCMV children. Larger numbers were used in a study of the effects of etiology on CI outcomes [37]. Nishio and colleagues collected clinical information from 308 pediatric cochlear implant cases, including etiology, hearing thresholds, age at CI, and early auditory skill development; of relevance, there were 24 children (7.8%) who were diagnosed with congenital CMV infection. As measured by IT-MAIS and MUSS, the genetic etiology group showed better development of hearing skills after a CI, followed by the unknown etiology group and finally the cCMV group. This finding is similar to ours, obtained in the cCMV group (children with and without comorbidities), since in our comparison group of no-cCMV children the etiology was genetic or unknown.

It should be emphasized that, after 1 year of CI use, the children with cCMV in our study group obtained an average LAEQ score of 29, which can be considered an age-appropriate level of auditory development [32]. There is ample evidence in the literature of a direct relationship between early auditory development and later development of language skills and social competence [38]. However, it is unknown if this is also the case for children with cCMV, as they might have more difficulty in developing more complex auditory skills. Considering the study’s limitation of relying solely on a parental questionnaire to assess early auditory development, a long-term follow-up study is needed to objectively assess speech and language development in CI children with cCMV.

## 5. Conclusions

In children with sensorineural hearing loss and congenital cytomegalovirus (CMV) infection who do not have comorbidities, early cochlear implantation leads to auditory development levels comparable to those observed in children without cCMV infection. Conversely, children with cCMV infection and comorbidities exhibit lower levels of auditory development compared to children without cCMV-related hearing loss.

## Figures and Tables

**Table 1:** Characteristics of the study group (with cCMV) and the reference group (no cCMV).

	cCMV Group		No cCMV Group	
	*N*	%	*N*	%
Gender				
Female	18	38.3	57	48.7
Male	29	61.7	60	51.3
CI ear				
Right	38	80.9	99	84.6
Left	9	19.1	18	15.4
Etiology of hearing loss				
Congenital cCMV	47	100.0		
Genetic			55	47.0
Unknown			48	41.0
Intrauterine infection other than cCMV			6	5.1
Prematurity			4	3.4
Ototoxic drugs			1	0.9
Meningitis			3	2.6
Comorbidities				
Congenital heart defect	1	2.1		
Cerebral palsy	5	10.6		
Epilepsy	5	10.6		
Delayed psychomotor development	9	19.1		
Fetal hypotrophy	3	6.4		
Congenital CNS disorder	2	4.3		
No comorbidities	22	46.8	117	100.0

## Data Availability

The data that support the findings of this study are available from the corresponding authors [PHS and AK] upon reasonable request.
